# Multi‐Dimensional Mechanical Mapping Sensor Based on Flexoelectric‐Like and Optical Signals

**DOI:** 10.1002/advs.202301214

**Published:** 2023-04-20

**Authors:** Peng Zhang, Zhaowei Teng, Lei Zhao, Zhichao Liu, Xue Yu, Xiaodie Zhu, Songcheng Peng, Ting Wang, Jianbei Qiu, Qingyuan Wang, Xuhui Xu

**Affiliations:** ^1^ Faculty of Materials Science and Engineering Key Laboratory of Advanced Materials of Yunnan Province Kunming University of Science and Technology Kunming 650093 P. R. China; ^2^ The Central Laboratory and Department of Orthopedic The Second Affiliated Hospital of Kunming Medical University Kunming 650106 P. R. China; ^3^ Clinical Medical Research Center and Key Laboratory of Yunnan Provincial Innovative Application of Traditional Chinese Medicine The First Peoples Hospital of Yunnan Province Affiliated Hospital of Kunming University of Science and Technology Kunming 650034 P. R. China; ^4^ School of Physics and Opto‐Electronic Technology Collaborative Innovation Center of Rare‐Earth Optical Functional Materials and Devices Development Baoji University of Arts and Sciences Baoji 721016 P. R. China; ^5^ Pillar of Engineering Product Development Singapore University of Technology and Design Singapore 487372 Singapore; ^6^ School of Mechanical Engineering Institute for Advanced Study Chengdu University Chengdu 610106 P. R. China; ^7^ College of Materials and Chemistry & Chemical Engineering Chengdu University of Technology Chengdu 610059 P. R. China

**Keywords:** flexoelectric‐like effect, intelligent perception, mechano‐luminescence, optical signals

## Abstract

Mechanical sensors execute multi‐mode response to external force, which are cornerstones for applications in human–machine interactions and smart wearable equipments. Nevertheless, an integrated sensor responding to mechanical stimulation variables and providing the information of the corresponding signals, as velocity, direction, and stress distribution, remains a challenge. Herein, a Nafion@Ag@ZnS/polydimethylsiloxanes (PDMS) composite sensor is explored, which realizes the description of mechanical action via optics and electronics signals simultaneously. Combined with the mechano‐luminescence (ML) originated from ZnS/PDMS and the flexoelectric‐like effect of Nafion@Ag, the corresponding explored sensor achieves the detection of magnitude, direction, velocity, mode of mechanical stimulation, and the visualization of the stress distribution. Moreover, the outstanding cyclic stability, linearity response character, and rapid response time are demonstrated. Accordingly, the intelligent recognition and manipulation of a target are realized, which indicate a smarter human–machine interface sensing applied for wearable devices and mechanical arms can be expected.

## Graphical abstract

Based on the flexoelectric‐like response of the Nafion@Ag and ML characteristic of ZnS/PDMS, a multi‐dimensional mechanical mapping sensor is explored with outstanding performances in direction and velocity identification, stress distribution mapping under mechanical stimulation. The corresponding explored sensor is integrated for multifunctional recognition and intelligent manipulation of a target object, which would provide immense potential for intelligent industrial robotics.

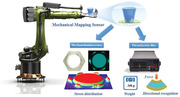

## 1 Introduction

One of the most indispensable tentacles for the perception of external information is mechanical sensor,^[^ ^1^ ^]^ which has been applied for intelligent robots,^[^ ^2^ ^]^ human health monitors,^[^ ^3^ ^]^ electronic skin,^[^ ^4^ ^]^ and haptic feedback,^[^ ^5^ ^]^ wearable smart healthcare.^[^ ^6^ ^]^ Mechanical sensors with electrical response signals based on resistive, capacitive, triboelectric, piezoelectric, and mechanical–potential change possess high sensitivity, wide detection range, and cost‐saving advantages.^[^ ^7^ ^]^ Nevertheless, conventional electrical–mechanical sensing systems is still facing the dilemma for the limited explored corresponding materials, complicated designs, and cumbersome fabrication processes. It aggravates the difficulty of multiple perceptual capability, and incompatible for the integrated and multifunctional detection of mechanical information.^[^ ^8^ ^]^ More recently, approaches to realize the multi‐dimensional censoring have been proposed, such as circuit integration, wireless technology, and self‐powered.^[^ ^9^ ^]^ Flexoelectric materials can realize the perception of direction and velocity while ensure the high sensitivity of the detected mechanical stimulation,^[^ ^10^ ^]^ whereas it remains challenging for dynamic visual detection, which is significant for human–machine interaction and intelligent sensing.^[^ ^11^ ^]^

mechano‐luminescence (ML) has become an emerging technique for stress sensing because of visual stress distribution, remote sensing, dynamic response, and a high correlation between ML intensity and mechanical amplitude.^[^ ^12^ ^]^ In the past decade, significant progresses on the research of ML have been achieved. Zhonglin Wang et al. developed a full dynamic‐range pressure sensor matrix that can accurately detect and spatially map pressure profiles by integrating an electrode triboelectric nanogenerator with the ML process.^[^ ^13^ ^]^ Dengfeng Peng et al. designed various heterojunctioned ZnS/CaZnOS and CaZnOS‐ZnS‐SrZnOS piezophotonic systems for the smart wearable flexible self‐luminous and printable devices.^[^ ^14^ ^]^ Yixi Zhuang et al. designed a novel stress distribution sensor with the detection sensitivity enhanced by two orders of magnitude thanks to the combination of the proposed near‐distance ML imaging scheme with an improved mechano‐to‐photon converter.^[^ ^15^ ^]^ Although the poor sensitivity and faint signal conversion of the as‐explored ML still need to be further improved, combining the multi‐response behavior of the ML with flexoelectric characteristic seems to provide a new perspective for mechanical detection.

Herein, we demonstrate an integrated multi‐dimensional mechanical mapping sensor based on Nafion@Ag@ZnS/PDMS composite sensor. The sensor combines the high sensitivity, orientation recognition capability based on the flexoelectric‐like response and visual stress distribution capability. Furthermore, the sensor has been integrated for the intelligent recognition and manipulation of the target object. Accordingly, the proposed multi‐dimensional mechanical mapping sensor could be prospective candidate in the fields of industrial robots, bionic sensing, automatic medical devices, and so on.

## 2 Results and Discussion

**Figure 1:**
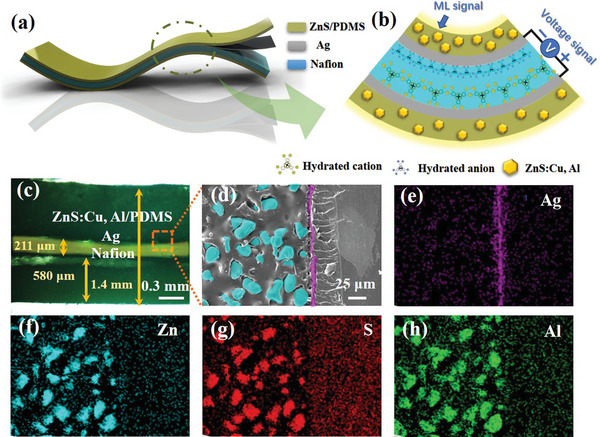
Schematic illustration of the NAZP sensor. a) Structure, b) mechanisms, c) micrograph, and d) SEM images of the NAZP sensor. EDS mapping images of e) Ag, f) Zn, g) S, and h) Al, respectively.

The structure and mechanisms of mechanical sensor are illustrated in **Figure** **1**. Figure 1a depicts the corresponding obtained sensor is composed of the Nafion substrate, Ag interlayers and ZnS/PDMS outer layers for the electrical signal sensing, electron migration, and optical signal sensing, respectively. As shown in Figure 1b, the differently sized hydrated metal cations (Na^+^) and anions are doped inside an elastomeric Nafion film. Upon bending the polymer film, cations and anions are separated due to the volumetric asymmetry in the anode and cathode, and then result in the generation of the potential difference based on flexoelectric‐like effect.^[^ ^10^, ^16^ ^]^ Furthermore, the ZnS particles generate visible ML signals by the activator recombination in association with local piezoelectric fields thanks to the induced de‐trapping of carriers at shallow traps under the external mechanical stimulation.^[^ ^12^, ^13^, ^17^ ^]^ Moreover, the micrograph of Nafion@Ag@ZnS/PDMS (NAZP) in Figure 1c,d reveals the sandwich‐like structure. The innermost layer is Nafion (211 µm thickness) used to generate the electrical signals, which is transmitted to the outside through the intermediate silver electrode layer (13 µm thickness, Figure S2, Supporting Information), and the outermost layer is the PDMS layer embedded with the homogeneous distributed ZnS particles to generate the ML signals (580 µm thickness). The energy dispersive X‐ray spectroscopy images energy dispersive spectrum is adopted to investigate the elementary composition of Ag and ZnS, as shown in Figure 1e–h and Figure S3 (Supporting Information), indicating that the uniform distribution of the elements of Ag, Zn, S, and Al, respectively. The XRD patterns of the as‐obtained ZnS: 0.05%Cu, 3%Al samples indicate the wurtzite phase (Figure S4, Supporting Information), and insignificant phase change is observed after doping with Cu and Al ions, accordingly.

**Figure 2:**
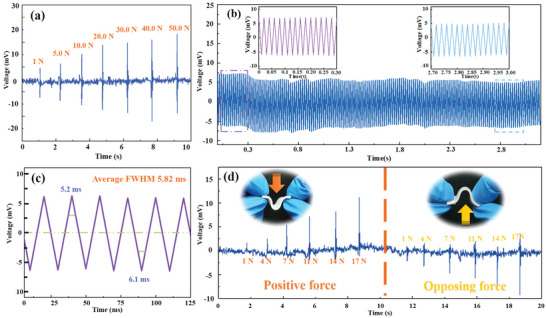
Electrical response performance of the explored NAZP mechanical sensor. a) Voltage‐time dynamic response performance with the reciprocating force varying from 0 to 50 N. b) Cyclic stability test for the sensor bent by >300 cycles. c) Response time under dynamic loaded force. d) Voltage‐time dynamic response performance in different directional and varying load from 0 to 17 N.

The mechanical–electrical response performance of the explored NAZP sensor is revealed in **Figure** **2**. Figure 2a presents the electrical signals strength response to varied mechanical stimulation, which reveals the feature of voltage increased with the loading magnitude. In the consideration of the deformation limits of the corresponding film, the voltage signal reaches the maximum when the applied load is up to 20 N. For long‐term service life, the current of the NAZP sensor <5 N is recorded in Figure 2b, which shows an insignificant disturbance over the whole period (300 cycles). The signal waveform of the NAZP sensor is identical in the first and last test periods (the insets in Figure 2b), confirming the excellent stability for the structural rigidity of the sandwich‐like structure. Furthermore, the response time is monitored in Figure 2c, which is defined as the full width at half‐maximum (FWHM) of an electrical signal. The results of response time from 100 measurements indicate a mean value of 5.82 ms, which indicates that the information under mechanical stimulation can perceived faster than the previously explored mechanical sensors (Table S1, Supporting Information). We attribute this phenomenon mainly to the rapid movement of hydration cations within the Nafion film. Then, the direction recognition ability in dynamic loads of the sensor is evaluated, as shown in Figure 2d. The voltage signals reveal positive and negative values in different directions, and the record signals enhance with the loads. The positive and negative voltage value is caused by the movement of hydrated cations in the Nafion film due to the different deformation directions.

**Figure 3:**
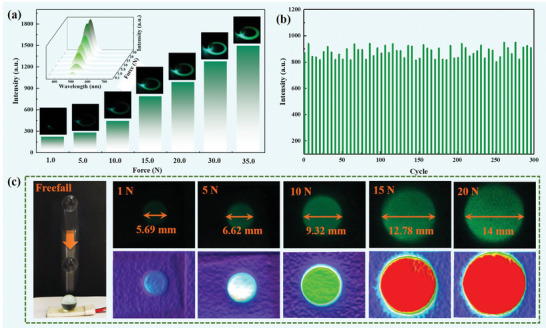
Optical‐response performance of the NAZP mechanical sensor. a) ML dynamic response performance under the load from 0 to 35 N. b) Cyclic stability test of the mechanical sensor by 300 cycles <20 N loaded force. c) Visualized stress distribution recorded for the ball‐dropping experimental.

Moreover, the mechanical–optical response ability of the sensor is measured, as shown in **Figure** **3**. Figure 3a presents the ML performance of the as‐fabricated mechanical sensor with various applied forces. The inset in Figure 3a is the corresponding ML spectra. The ML intensity of sensors increases with applied load, which is attributed to the piezoelectricity strengthening response to the load.^[^ ^12^, ^14^ ^]^ The NAZP sensor exhibits excellent operating stability at 20 N for 300 cycles owing to its sturdy construction, as shown in Figure 3b. The dynamic stress mapping of the NAZP sensor was tested by a ball‐dropping experiment at different heights (Figure 3c). ML mappings of different magnitudes were generated when the falling ball crashed the NAZP sensor. By extracting and analyzing the grayscale of the captured images, a visual stress distribution of the light intensity could be obtained, as shown at the bottom of Figure 3c. Herein, the explored sensor can intuitively, visually detect and sense the stress distribution, which exhibits promising prospects for human–machine interaction and the intelligence electronic skin.

**Figure 4:**
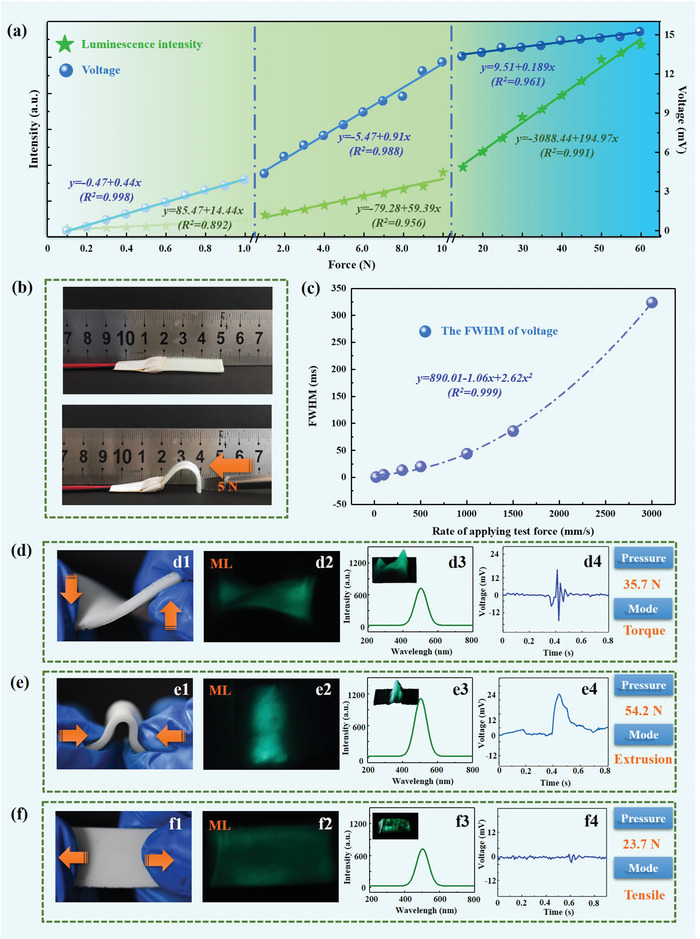
The comprehensive properties of the NAZP mechanical sensor under different force magnitude, velocity, and modes of mechanical stimulation. a) Optical‐response and electro‐response performance of the sensor with the force varying from 0 to 60 N. b) The evaluation of the velocity of the upload force. c) Electro‐response of sensor under the different velocity. Optical‐response and electro‐response of the explored sensor under d) torque, e) extrusion, and f) tensile of force stimulation, respectively.

Herein, we further investigated the performance of the explored NAZP sensor under various loads, velocity, and complex mechanical stimulations. The plotted ML intensity and voltage versus the applied force of the NAZP sensor assessed in **Figure** **4**a suggests the outstanding linear dependence of the ML intensity and voltage on the imposed force. The response linearity of the optical and electrical signals is calculated to be *R* ^2^_electric_ = 0.998/*R* ^2^_optical_ = 0.892, *R* ^2^_electric_ = 0.988/*R* ^2^_optical_ = 0.985, and *R* ^2^_electric_ = 0.961/*R* ^2^_optical_ = 0.991 under the loading range of 0–1, 1–10, and 10–60 N, respectively. The distinct linear dependence behavior of the optical and electrical signals suggests the respective advantages to the uploaded force range, which could recognize the magnitude of the applied loads through the linear dependence more precisely. Then, the velocity perception ability of the sensor was evaluated in Figure 4b, and the corresponding FWHM value of the electrical signals is plotted in Figure 4c. We noticed that the FWHM values of voltage signals are highly correlated with the velocity of the uploaded force ranging from 0 to 3000 mm s^−1^, exhibiting excellent sensitivity. It is directly related to the velocity of the Nafion deformation, which affects the charge transfer in the film, accordingly. Furthermore, the ability of the sensor to recognize complex mechanical stimulations is depicted, including the torque (Figure 4d1), extrusion (Figure 4e1), and tensile (Figure 4f1) of the uploaded force, respectively. The visual detection of the uploaded force through ML distribution images is presented in Figure 4d2,e2,f2, respectively, and the magnitude of mechanical loads and velocity through the linear dependence of the ML intensity is deduced Figure 4d3,e3,f3. Moreover, the voltage signals response to the imposed force is displayed in Figure 4d4,e4,f4, respectively. The sense direction is described with the positive and negative voltage signals. Herein, determining the stress mode of mechanical stimulation by comprehensive analysis of signal differences could be achieved.

**Figure 5:**
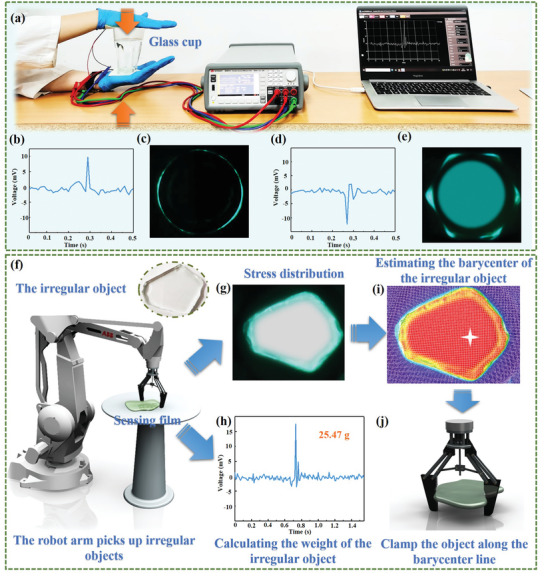
NAZP mechanical mapping sensor for user‐interactive applications. a) The integration of intelligent gloves and an experimental demonstration of grasping glass cup. The visual pattern of electrical response signals and optical stress distribution of the gloves on the upper b,c) and lower d,e) glass cups. f–j) The concept of the NAZP mechanical mapping sensor assisted the intelligent robots to recognize and grasp an irregular object. Contour recognition g) and weight detection h) of an irregular object by visualizing stress distribution images and electrical signal responses, respectively. i) The barycenter of the object calculated by using the open CV algorithm to process the stress distribution image. j) The grasping action performed by the intelligent robot.

As a proof of concept experiment, the integration of NAZP mechanical mapping sensors into gloves represents a promising paradigm for user‐interactive interfaces, as displayed in **Figure** **5**a. When the gloves containing the sensors touch the glass cup, the direction and weight of the cup can be accurately perceived through electrical signals (Figure 5b–d) to assist the intelligent grasping action of the robot arm. Besides, the profile of the glass cup can be visually distinguished by the stress mapping images (Figure 5c,e), which enables convenient interaction between wearers and supervisors. Considering the distinctive performance of NAZP mechanical mapping sensors, assisting the intelligent robotic arm to recognize and grasp an irregular object is further realized (Figure 5f). First, the intelligent robot equipped with NAZP mechanical mapping sensors can visually distinguish the profile of irregular objects by stress mapping images (Figure 5g), and accurately detect the weight of the irregular object (Figure 5h) through converting the feedback electrical signal using the correlation formula in Figure 5a. Then, the barycenter of the object is calculated by using the open CV algorithm (an open‐source computer vision library) to process the stress distribution image (Figure 5i). Finally, the intelligent robot could quickly and accurately grasp the object along the barycenter line after completing the overall perception of the irregular object (Figure 5j). It would greatly simplify the traditional robot grasping process, integrated with the perception of the object's weight, direction, and contour information, which holds immense potential in intelligent robot and human–machine interfacing.

## 3 Conclusion

In summary, based on the flexoelectric‐like response of the Nafion@Ag and ML characteristic of ZnS/PDMS, a multi‐dimensional mechanical mapping sensor is explored with the outstanding performances in direction and velocity identification, and stress distribution mapping under mechanical stimulation. Moreover, cyclic stability, excellent linearity (*R* ^2^>0.99), and fast response time (5.82 ms) are unambiguously demonstrated. The corresponding explored sensor has been integrated for multifunctional recognition and intelligent manipulation of a target object, which would provide immense potential as a self‐powered sensor for human–machine interfaces, next‐generation wearable electronics, and intelligent industrial robotics in the future.

## 4 Experimental Section

### Materials

Nafion 117 film was purchased from DuPont Company. Hydrogen peroxide solution (H_2_O_2_, 30wt.%), sodium hydroxide (NaOH), ammonia solution (NH_4_OH), glucose (C_6_H_12_O_6_), silver nitrate (AgNO_3_), cupric nitrate (Cu(NO_3_)_2_·3H_2_O), zinc sulfide (ZnS), aluminum oxide (Al_2_O_3_) concentrated hydrochloric acid (HCl, 36–38wt.%), and Polydimethylsiloxane were supplied by Aladdin Company.

### Fabrication of the Nafion@Ag (Ionic Polymer Metal Composite, IPMC)

First, the Nafion 117 film was sanded with 600 grit sandpapers to form a rough groove on the surface. Before the fabrication of the Ag electrodes, the Nafion 117 film was treated with several procedures, including the immersion within a 15 wt.% H_2_O_2_ aqueous solution at 95°C for 30 min, boiled in deionized water at 100°C for 30 min, dipped in a 2 mol L^−1^ HCl aqueous solution for 40 min, boiled again in deionized water at 100°C for 30 min, and subsequently in a 0.25 mol L^−1^ NaOH at 30°C for 30 min. Then, the Nafion 117 film was impregnated into a 0.03 mol L^−1^ Tollens' reagent solution (100 mL), which was chemically precast by NH_4_OH and AgNO_3_ H_2_O for 15 h to allow the sufficient impregnation of Ag^+^ ions. Finally, the C_6_H_12_O_6_ solution 20 mL of 0.15 mol L^−1^ was slowly dropped into the previous solution, the C_6_H_12_O_6_ was able to reduce Ag^+^ ions to be Ag elementary substance which was deposited on the surface of the Nafion film. This impregnation/reduction step was repeated three times to get a better interface fastness and surface conductivity of the Ag electrode.

### Fabrication of the ZnS: Cu, Al

ZnS: 0.05%Cu, 3%Al particles were synthesized by a high temperature solid‐state reaction at atmospheric pressure. ZnS, Cu(NO_3_)_2_·3H_2_O, and Al_2_O_3_ were mixed in a certain proportion by wet grinding. The mixture was compacted into an alumina boat and then sintered in furnace at 1100°C for 3 h. Finally, the products were ground and screened to characterize them.

### Fabrication of the Nafion@Ag@ZnS/PDMS (NAZP) Sensor

The fabrication process of the mechanical sensing sensor was shown in Figure S1 (Supporting Information). The liquid PDMS and ZnS: Cu, Al were mixed evenly in a ratio of 2:1. The above mixture was poured into a glass mold with the length, width, and thickness of 30 mm × 10 mm × 3 mm. Copper wires were connected on both sides of the Nafion@Ag film for electrical signal detection. Afterward, the Nafion@Ag film (25 mm×10 mm) with copper wires was wrapped by the as‐obtained ZnS: Cu, Al/PDMS mixture solution. It was degassed in a vacuum oven for 10 min. Finally, the NAZP sensor of sandwich‐like structure was prepared after being cured and dried in an oven at 80°C for 6 h.

### Material Characterization

The phase samples were identified via XRD measurement (D8ADVANCE/Germany Bruker X‐ray diffractometer), with Cu‐Ka radiation (l = 0.15405 nm) in the 2*θ* range from 10°–70°. The microstructure of the NAZP was analyzed by optical microscope (ECLIPSE Ti2, Nikon) and SEM (JIB‐4700F). Sample morphology and the energy‐dispersive spectrum (EDS) were measured using a scanning electron microscope (JIB‐4700F). ML signals were collected in situ from a rotary friction testing machine (MS‐T3001) to a fluorescence spectrophotometer (QP600‐025‐VIS‐NIR, Ocean Optics). The electrical response signals were measured by Source‐Measure Unit equipment (Keysight B2900A).

## Conflict of Interest

The authors declare no conflict of interest.

## Supporting information

Supporting Information

## Data Availability

Research data are not shared.
