# Supplementary material for: Multi‐Dimensional Mechanical Mapping Sensor Based on Flexoelectric‐Like and Optical Signals
Source: Adv Sci (Weinh). 2023 Apr 20;10(19):2301214. doi: 10.1002/advs.202301214 (PMC10323605; doi:10.1002/advs.202301214)
Supplement: Supplementary file 1 — Supporting Information [file ADVS-10-2301214-s001.pdf]

ADVANCED SCIENCE
Open Access

Supporting Information

for *Adv. Sci.*, DOI 10.1002/advs.202301214

Multi-Dimensional Mechanical Mapping Sensor Based on Flexoelectric-Like and Optical Signals

Peng Zhang, Zhaowei Teng, Lei Zhao, Zhichao Liu, Xue Yu, Xiaodie Zhu, Songcheng Peng, Ting Wang*, Jianbei Qiu, Qingyuan Wang and Xuhui Xu**

Supporting Information

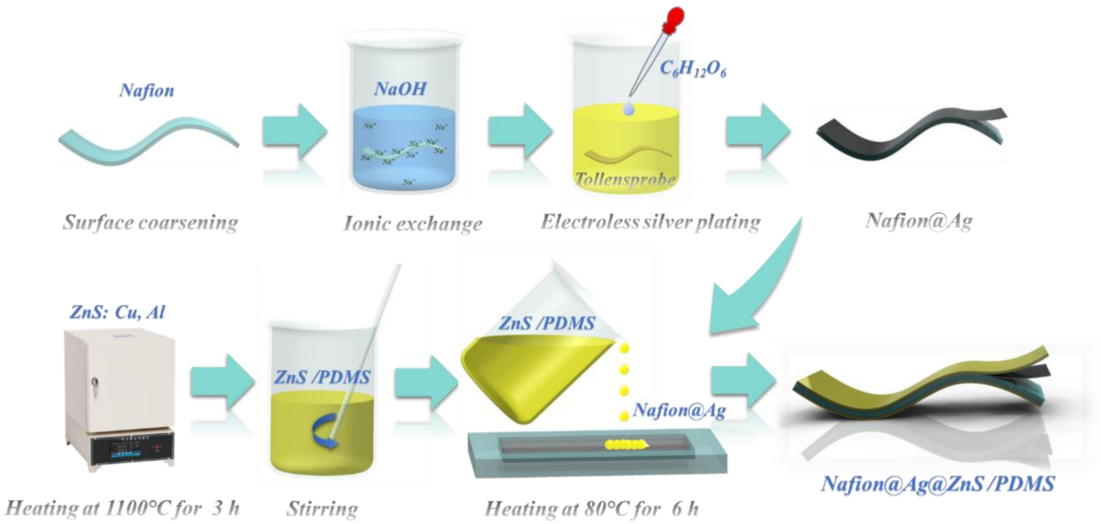

Figure S1. Schematic diagram of the preparation process of Nafion@Ag@ZnS/PDMS composite film.

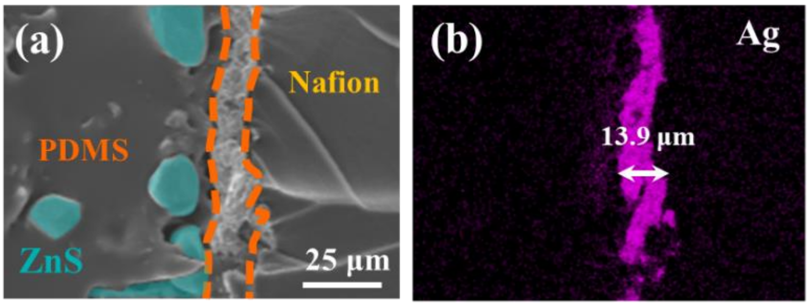

Figure S2. The enlarged SEM images of Ag layer.

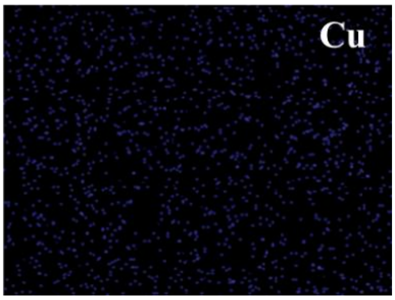

Figure S3. The elemental mapping image of Cu.

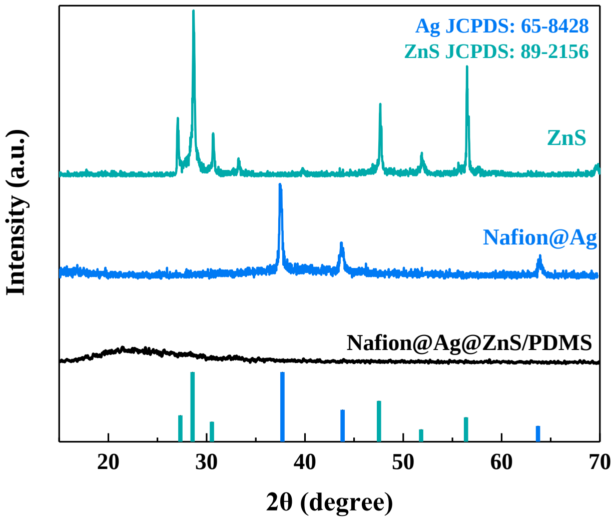

Figure S4. XRD patterns of Nafion@Ag, ZnS, and Nafion@Ag@ZnS/PDMS film, respectively.

Table S1. Summary of mechanical sensors and their performance parameters.

Materials	Device Type	Sensor detection information	Response time	Cyclic stability	Response linearity	References
Nafion@Ag@ZnS/PDMS	Flexoelectric-like and ML	Magnitude of force, Direction, Velocity, Mode, Stress distribution	5.82 ms	＞300	R^2=0.99	This work
CB/Mxene/PDMS composites	Rriboelectric signals	Differentiate the normal and shear forces	0.26 s	28000	R^2 = 0.9773	[1]
Laser-induced graphene/PDMS	Electromechanical	Sportswear	210 ms	30000	R^2=0.99	[2]
ZnS−CaZnOS	ML	Temperature and pressure		2300/8000	R^2=0.99	[3]
Pressure-sensitive conductive sheet/FPCB	Piezoelectricity	Six-dimensional force perception			linearity error ＜0.008	[4]
Ag/SiO_2; ZnS:Mn/PDMS	ML and triboelectric	Full dynamic-range pressure	9.23 ms		linear relationship (the slope is 0.029 MPa^{-1})	[5]
Polymer gel	Piezoionic mechanisms	Force 0.01 to 100 nV/Pa	2 ms			[6]
3D capacitive sensor with a seesaw-like shape	Capacitive sensor	Force and acceleration measurement		50	approximate linear correlation	[7]
A carbon flower	Resistance	Pressure sensing		1000	0.025 N	[8]

and elastomer composite						
Pyramid microstructured dielectric layer	Capacitive	Pressure sensors	25 μm	2000	pulsations at 43 bpm	[9]
Biodegradable materials	Piezoelectricity	Strain sensor	A response time in the millisecond range	30000	0.89	[10]
Flexible sensing arrays (PDMS /Ni-Au)	Piezoelectricity	Strain sensor	25 ms	4000		[11]
Nacre-mimetic multi-layered silver nanowires /reduced graphene oxide /thermoplastic polyurethane mats	Piezoresistance	Contact-Noncontact Sensing	20 ms	11000	0.967	[12]
ZnS:Mn	ML	personalized pressure information	10 ms	＞10000	obvious linearly increasing trend	[13]

References

[1] W. Zhang, Y. Xi, E. Wang, X. Qu, Y. Yang, Y. Fan, B. Shi, Z. Li, *ACS Appl Mater Interfaces* **2022**, *14* (17), 20122.

[2] T. Raza, M. K. Tufail, A. Ali, A. Boakye, X. Qi, Y. Ma, A. Ali, L. Qu, M. Tian, *ACS Appl Mater Interfaces* **2022**, *14* (48), 54170.

[3] X. Ma, C. Wang, R. Wei, J. He, J. Li, X. Liu, F. Huang, S. Ge, J. Tao, Z. Yuan, P. Chen, D. Peng, C. Pan, *ACS Nano* **2022**, *16* (2), 2789.

[4] J. H. Hu, Y. Qiu, X. Wang, L. L. Jiang, X. Y. Lu, M. Li, Z. Q. Wang, K. Pang, Y. Tian, W. N. Zhang, Z. Xu, H. J. Zhang, H. C. Qi, A. P. Liu, Z. Zhang, H. P. Wu, *Nano Energy* **2022**, *96*, 107073.

[5] X. Wang, M. Que, M. Chen, X. Han, X. Li, C. Pan, Z. L. Wang, *Adv Mater* **2017**, *29* (15).

[6] Y. Dobashi, D. Yao, Y. Petel, T. N. Nguyen, M. S. Sarwar, Y. Thabet, C. L. W. Ng, E. Scabeni Glitz, G. T. M. Nguyen, C. Plesse, F. Vidal, C. A. Michal, J. D. W. Madden, *Science* **2022**, *376* (6592), 502.

[7] J. L. Ye, F. Zhang, Z. M. Shen, S. Z. Cao, T. Q. Jin, X. G. Guo, Z. H. Li, L. Lin, Y. H. Zhang, *Npj Flexible Electronics* **2021**, *5* (1), 2397.

[8] S. J. K. O'Neill, H. X. Gong, N. Matsuhisa, S. C. Chen, H. Moon, H. C. Wu, X. F. Chen, X. D. Chen, Z. N. Bao, *Adv Mater Interfaces* **2020**, *7* (18).

[9] S. R. A. Ruth, L. Beker, H. Tran, V. R. Feig, N. Matsuhisa, Z. A. Bao, *Advanced Functional Materials* **2020**, *30* (29).

[10] C. M. Boutry, Y. Kaizawa, B. C. Schroeder, A. Chortos, A. Legrand, Z. Wang, J. Chang, P. Fox, Z. N. Bao, *Nature Electronics* **2018**, *1* (5), 314.

[11] Y. X. Wan, J. Tao, M. Dong, L. Zhang, Z. C. Peng, R. R. Bao, C. F. Pan, *Advanced Materials Technologies* **2022**, *7* (12).

[12] K. Zhou, W. Xu, Y. Yu, W. Zhai, Z. Yuan, K. Dai, G. Zheng, L. Mi, C. Pan, C. Liu, C. Shen, *Small* **2021**, *17* (31), e2100542.

[13] X. Wang, H. Zhang, R. Yu, L. Dong, D. Peng, A. Zhang, Y. Zhang, H. Liu, C. Pan, Z. L. Wang, *Adv Mater* **2015**, *27* (14), 2324.
